# Acidosis Mediates the Switching of G_s_-PKA and G_i_-PKC_ε_ Dependence in Prolonged Hyperalgesia Induced by Inflammation

**DOI:** 10.1371/journal.pone.0125022

**Published:** 2015-05-01

**Authors:** Wei-Yu Huang, Shih-Ping Dai, Yan-Ching Chang, Wei-Hsin Sun

**Affiliations:** 1 Department of Life Sciences, National Central University, Jhongli, Taiwan; 2 Institute of Systems Biology & Bioinformatics, National Central University, Jhongli, Taiwan; University of South Alabama, UNITED STATES

## Abstract

Chronic inflammatory pain, when not effectively treated, is a costly health problem and has a harmful effect on all aspects of health-related quality of life. Previous studies suggested that in male Sprague Dawley rats, prostaglandin E_2_ (PGE_2_)-induced short-term hyperalgesia depends on protein kinase A (PKA) activity, whereas long-lasting hyperalgesia induced by PGE_2_ with carrageenan pre-injection, requires protein kinase C_ε_ (PKC_ε_). However, the mechanism underlying the kinase switch with short- to long-term hyperalgesia remains unclear. In this study, we used the inflammatory agents carrageenan or complete Freund’s adjuvant (CFA) to induce long-term hyperalgesia, and examined PKA and PKC_ε_ dependence and switching time. Hyperalgesia induced by both agents depended on PKA/PKC_ε_ and G_s_/G_i_-proteins, and the switching time from PKA to PKC_ε_ and from G_s_ to G_i_ was about 3 to 4 h after inflammation induction. Among the single inflammatory mediators tested, PGE_2_ and 5-HT induced transient hyperalgesia, which depended on PKA and PKC_ε_, respectively. Only acidic solution-induced hyperalgesia required G_s_-PKA and G_i_-PKC_ε_, and the switch time for kinase dependency matched inflammatory hyperalgesia, in approximately 2 to 4 h. Thus, acidosis in inflamed tissues may be a decisive factor to regulate switching of PKA and PKC_ε_ dependence via proton-sensing G-protein–coupled receptors.

## Introduction

Tissue injury, infection or tumor growth induces inflammation, which is often accompanied by persistent and chronic pain. The production and release of inflammatory mediators (e.g., protons, prostaglandin E_2_ [PGE_2_], serotonin [5-hydroxytryptamine (5-HT)], bradykinin [BK], adenosine triphosphate) from the primary sensory terminal and non-neural cells in the inflamed sites heighten the pain experience by increasing the sensitivity of nociceptors to both thermal and mechanical stimuli [1,2].

Earlier studies of single inflammatory mediators demonstrated that BK, PGE_2_, 5-HT, and protons have excitatory action on cutaneous nociceptors and induce transient pain [3–6]. More sustained pain effects are achieved only with high concentration (10^-5^ M) of a combination of inflammatory mediators (including BK, 5-HT, PGE_2_, and histamine)[7]. High local proton concentrations in inflamed tissues excite and sensitize rat skin nociceptors and cause sustained pain in human skin [5,8,9]. The combination of inflammatory mediators (BK, 5-HT, PGE_2_, and histamine) in acid solution (pH 6.1) excites and sensitizes rat skin nociceptors [10]. Steen et al. [11] proposed that a combination of inflammatory mediators plays a role in sensitizing the low pH effect. A proton-activated sustained current is potentiated stronger by a combination of mediators than each mediator alone [12]. Accordingly, acidosis in inflamed tissues is the decisive factor for ongoing nociceptor excitation and sustained pain [13].

Administration of epinephrine induces short-term hyperalgesia, which depends on protein kinase A (PKA) and protein kinase Cε (PKCε) activity [14,15], whereas PGE_2_-induced short-term hyperalgesia depends on only PKA activity [16]. With carrageenan pre-injection before PGE_2_, rats display long-lasting hyperalgesia and the prolonged effect can be inhibited by a PKCε blocker or attenuated by antisense oligonucleotides for PKCε [17,18]. Therefore, PKCε is necessary to maintain hyperalgesic priming. Parada et al. [19] proposed that PKCε-mediated hyperalgesic priming depends on cAMP. The cAMP-dependent PKCε activation is probably through Epac [20]. In contrast, Gi-mediated pathway is also suggested to participate in PKCε activation in other studies [21–23]. Whether chronic inflammatory pain induced by inflammatory agents has a similar mechanism of the kinase switch remains unclear.

Here, we have demonstrated that both carrageenan and complete Freund’s adjuvant (CFA) conferred PKA- and PKCε-dependent hyperalgesia, and the switching time from PKA to PKCε was approximately 3 to 4 h after inflammation induction. Acidic solution-induced hyperalgesia also showed PKA and PKCε dependence, with the switch time at about 2 to 4 h. Acidosis in inflamed tissues is likely the major factor affecting PKA and PKCε dependence. Given that two proton-sensing G-protein—coupled receptors (GPCRs), G2A and TDAG8, were significantly increased after CFA injection, G2A and TDAG8 may mediate proton signals in the switch of PKA and PKCε.

## Materials and Methods

### Agents

The agents CFA, carrageenan, PGE_2_, 5-HT, pertussis toxin (PTX), U73122 (1-[6-[[(17b)-3-Methoxyestra-1,3,5(10)-trien-17-yl]amino]hexyl]-1H-pyrrole-2,5-dione), MES (2-(N-morpholino)ethanesulfonic acid), and HEPES (4-(2-hydroxyethyl)-1-piperazineethanesulfonic acid) were from Sigma. H89 dihydrochloride (N-[2-[[3-(4-Bromophenyl)-2-propenyl]amino]ethyl]-5-isoquinolinesulfonamide dihydrochloride), SQ22536 (9-(Tetrahydro-2-furanyl)-9H-purin-6-amine), and gallein (3’,4’,5’,6’-tetrahydroxyspiro[isobenzofuran-1(3H),9’-(9H)xanthen]-3-one) were from Tocris Bioscience. PKCεV_1-2_ peptide conjugated with the protein transduction domain of TAT protein for membrane permeability [24] (CYGRKKRRQRRR-CEAVSLKPT, TAT-PKCεV_1-2_) and control peptides (CYGRKKRRQRRR, TAT) were a kind gift from KAI Pharmaceuticals (CA, USA). For animal experiments, all drugs or peptides were diluted into saline before injection.

### Animals

CD1/ICR mice (8–12 weeks old) were purchased from BioLASCO Taiwan (Taipei, Taiwan) and housed 3–4 per cage under a 12-h light/dark cycle (lights on at 7:00am) with food and water *ad libitum* in a temperature and humidity controlled environment at the National Central University. Care and use of mice conformed the Guide for the Use of Laboratory Animals (US National Research Council) and the experimental procedures were approved by the local animal use committee (IACUC, National Central University, Taiwan). All behavioural testing was performed between 9:00am and 5:00pm. Effort was made to minimize the number of animals used and their suffering. For gene expression, mice were placed in the euthanasia chamber and sacrificed by introducing 100% carbon dioxide with a fill rate of 20%-30%/min. Mice were unconscious usually within 2 to 3 minutes. After sacrifice, dorsal root ganglia (DRG) were taken for RNA extraction.

### Inflammation experiments and dorsal root ganglia (DRG) tissue collection

Mice received an intraplantar injection with 25 μl of saline, CFA (50% in saline) or carrageenan (20 mg/ml in saline). At 4 or 24 h after injection, mice were killed and paw thickness was measured. For gene expression experiments, lumbar 4–6 (L4-6) DRG ipsilateral and contralateral to injected paws were removed at 90 min or 4 or 24 h for RNA extraction, with the ganglia from uninjected paws as negative controls. For animal experiments, mechanical tests were performed after CFA or carrageenan injection. For experiments of single mediators, mice were intraplantarly injected with PGE_2_, 5-HT or different pH solutions (10 mM MES pH4.0, 5.0, 5.5 or 10 mM HEPES pH6.0, pH7.4), followed by mechanical tests. In some experiments, mice were injected with inhibitors before or after injection of CFA, carrageenan, or single mediators and mechanical tests were performed after the second injection.

### Behavioural tests

Pain behavioural tests were described previously [25]. Briefly, mice were injected with 25 μl CFA, carrageenan, single mediators, or inhibitors, then animals were tested for withdrawal thresholds to mechanical stimuli (von Frey filaments, Touch-Test, North Coast Medical, Morgan Hill, CA) applied to the hindpaw. Mice (n ≥6 per group) were pre-trained for 1 to 2 h each day for 2 days before the test. A series of von Frey fibers were applied onto the plantar surface of both hindpaws at certain times after injection. For each paw, a von Frey fiber was applied 5 times at 5-s intervals. The paw withdrawal threshold (PWT) was determined when paw withdrawal was observed in at least 3 of 5 applications (>50%).

### RNA preparation and quantitative RT-PCR

RNA extraction from DRG was performed as described [26]. Each DRG pool contained at least 9 to 12 DRG from 3 to 4 mice. RNA was extracted by use of the RNeasy kit (Qiagen, Valencia, CA). Gene primers (100 nM), derived cDNA, and master mix (SYBR green I and AmpliTaq Gold DNA polymerase [Applied Biosystems, Foster City, CA]) were mixed for PCR reactions and product detection by using ABI Prism 7300. For each assay, preparations were run in triplicate. The thermal cycling conditions were 95°C for 10 min, followed by 40 cycles of 95°C for 15 s, and 60°C for 1 min. The threshold cycle (Ct) values of both the targets and internal reference (mGAPDH) were measured from the same samples, and the expression of the target genes relative to that of mGAPDH was calculated by the comparative Ct method.

The primer sequence sets were for OGR1 (151 bp), 5'-gacgataccagcccaagtgt-3’ (forward) and 5'-gctgttatccctagccacca-3’ (reverse); GPR4 (199 bp), 5'-cttcctcagcttcccaagtg-3’ (forward) and 5'-cctgggcctcctttctaaac-3’ (reverse); G2A (166 bp), 5'-aagtgtccagaatccacacagggt-3’ (forward) and 5'-agtaaacctagcttcgctggctgt-3’ (reverse); and TDAG8 (197 bp), 5'-atagtcagcgtcccagccaac-3’ (forward) and 5'-cgcttcctttgcacaaggtg-3’ (reverse). The internal control was measured from the same samples [mGAPDH, NM_001001303, 233 bp, primers: 5'-ggagccaaacgggtcatcatctc-3’ (forward) and 5'-gaggggccatccacagtcttct-3’ (reverse)].

### Immunostaining and confocal microscopy

Mouse DRG were isolated and cultured as described [27]. Briefly, DRG were treated with 0.125% collagenase IA for 5 min, then 0.25% trypsin for 15 min. DRG was dissociated by trituration with a fire-polished Pasteur pipette and cultured in Dulbecco's modified Eagle medium supplemented with 10% fetal bovine serum at 37°C for 12 to 14 h.

DRG neurons were stimulated with acidic solution with the indicated pH HEPES/MES buffer (125 mM NaCl, 1 mM KCl, 5 mM CaCl_2_, 1 mM MgCl_2_, 8 mM glucose, 10 mM HEPES and 15 mM MES, pH 7.6, 6.4 and 5.5) for 5 or 30 min or 15 s at 37°C. Cells were then fixed with 4% paraformaldehyde at 4°C for 30 min. After being blocked with 1% bovine serum albumin (BSA) in phosphate buffer saline (PBS), cells were stained with the primary antibody anti-PKA (1:250) or anti-PKCε (1:250, both Santa Cruz Biotechnology), then secondary antibody, TRITC-conjugated goat-anti-rabbit IgG (1:250, Sigma). All antibodies were diluted in PBS containing 1% BSA. All incubations were performed at 4°C overnight.

The specimens were observed under a confocal microscope (Zeiss LSM510) equipped with 561-nm/DPSS 561–10 Laser and Zeiss Plan-Apo oil-immersion 100X objective lens. The images were captured by use of AxioVixion 4.8. The fluorescence intensity of neurons was measured by selecting a straight line across the neuron soma and using the plot profile function of the image software. The fluorescence intensity on the line (F_0_) was normalized by the averaged fluorescence intensity of the line (F_avg_). The peripheral region of the soma defined as the membrane region was 0 to 10% and 90% to 100% of the distance across the cell. The central region of the soma defined as the cytosol was 10% to 90% of the distance across the cell.

### Statistical analysis

Data are presented as mean±SEM. One-way or two-way ANOVA with post-hoc Bonferroni correction was used to compare multiple groups. P<0.05 was considered statistically significant.

## Results

### Prolonged hyperalgesia depends on a switch of PKA and PKCε kinase activities

**Fig 1:**
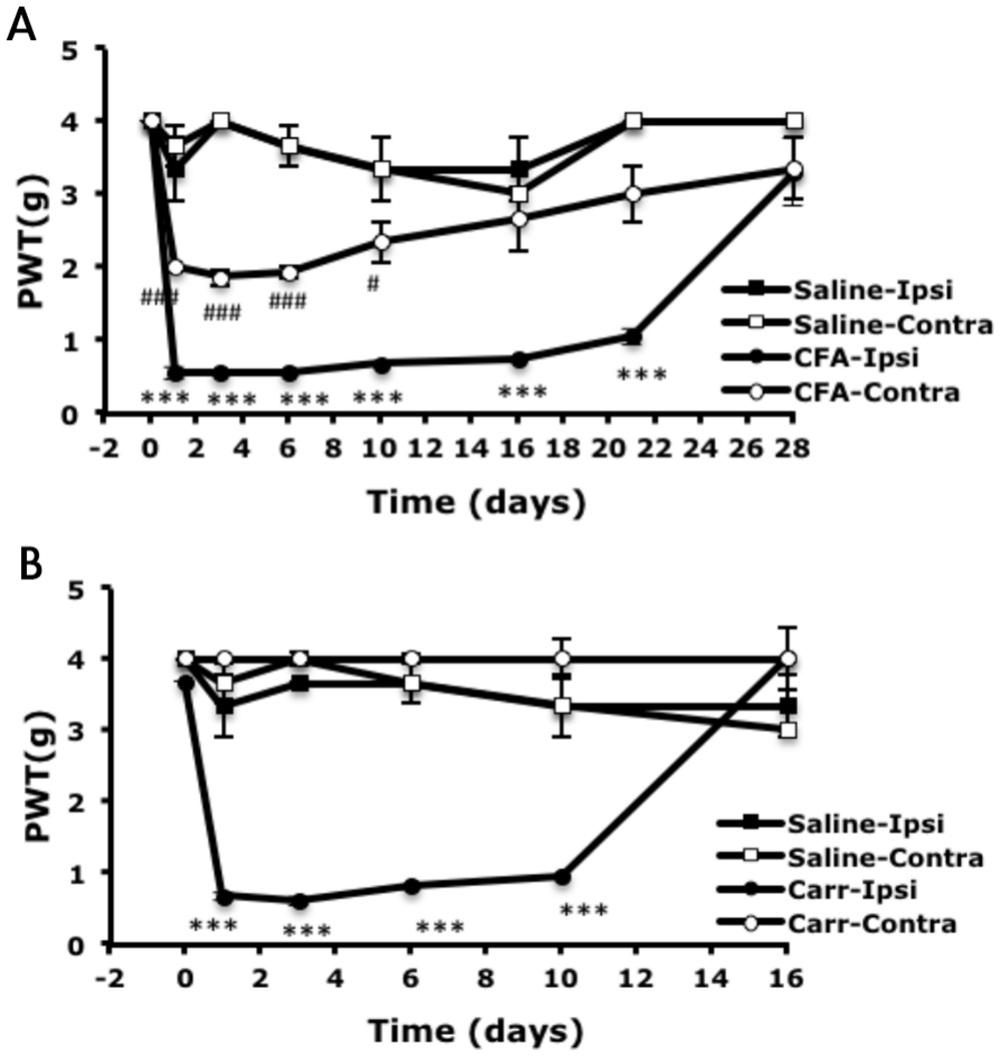
Mice show mechanical hyperalgesia with peripheral inflammation induced by complete Freund’s adjuvant (CFA) or carrageenan injection. Wild-type male CD1/ICR mice (8–12 weeks old) received intraplantar injection in the right hind paws with 25 μl CFA (50% in saline, A), carrageenan (Carr; 20 mg/ml, B), or saline (A, B). The threshold of paw withdrawal (PWT) to mechanical stimuli was measured before (t = 0) and after injection. Data are mean±SEM of total tested mice (n = 3–6 per group). ***p<0.001 compared to saline-injected ipsilateral group; ###p<0.001 compared to saline-injected contralateral group by two-way ANOVA with a post-hoc Bonferroni test.

Carrageenan and CFA are commonly used in models of inflammatory pain. Both induce prolonged hyperalgesia. To understand whether the prolonged hyperalgesia induced by these two agents also depends on PKA and/or PKCε, mice received intraplantar injection of CFA or carrageenan to induce peripheral inflammation. With 50% CFA injection, mice showed bilateral mechanical hyperalgesia at 30 min (0.73±0.04 and 2.33±0.67 g PWT on ipsilateral and contralateral paws, respectively; 3.67±0.67 g PWT with saline control) (Fig 1A). The hyperalgesia lasted for 21 days (0.6±0 g PWT on ipsilateral paws) and returned to basal level on day 28. Saline-injected mice did not show significant hyperalgesia in ipsilateral or contralateral sides. Mice injected with carrageenan also showed mechanical hyperalgesia at 30 min after injection (Fig 1B). As compared with CFA-induced hyperalgesia, carrageenan-induced hyperalgesia was unilateral (0.73±0.08 and 4±0 g PWT on ipsilateral and contralateral paws, respectively). The hyperalgesia remained for 10 days (0.93±0.01 g PWT on ipsilateral paws) and returned to baseline on day 16.

**Fig 2:**
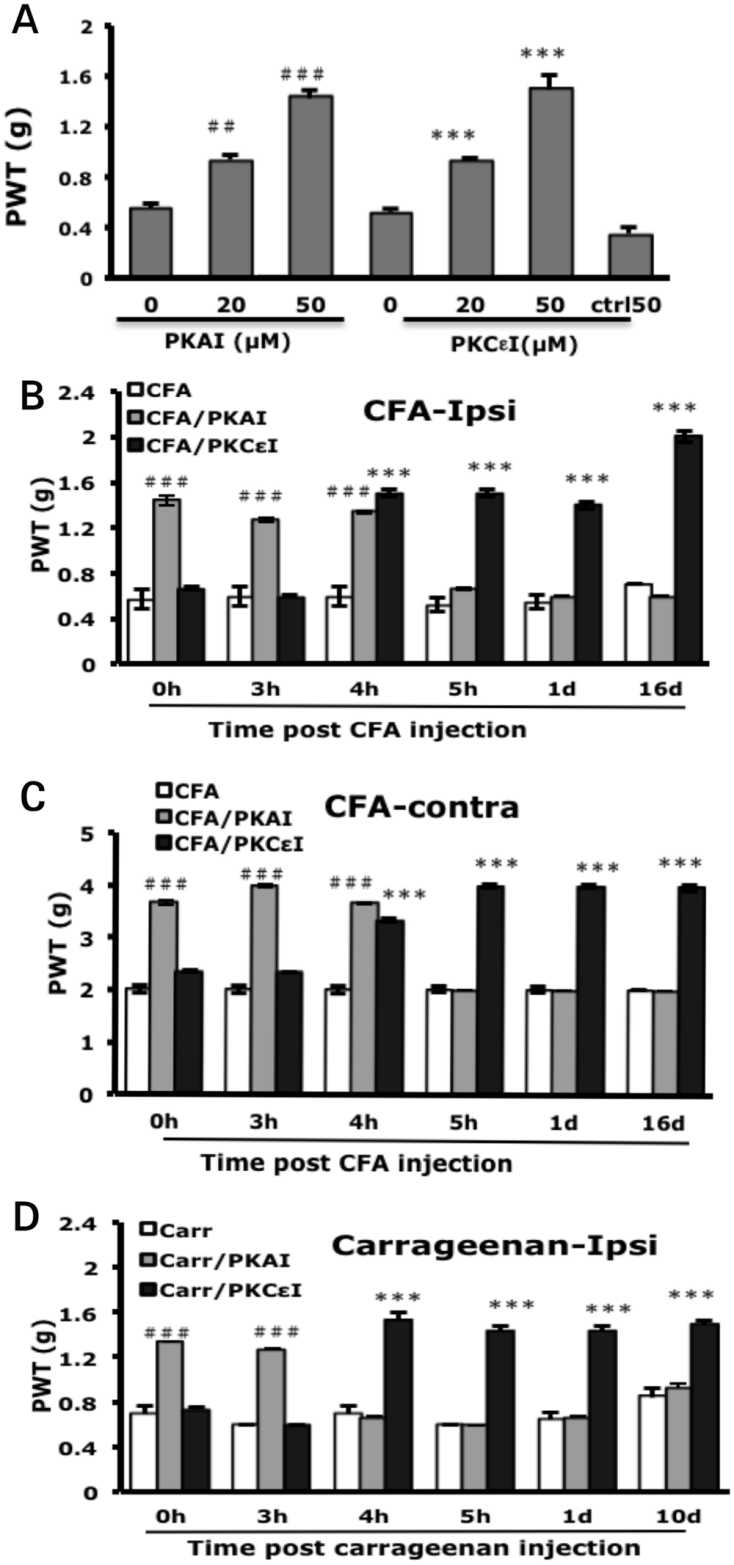
CFA- or carrageenan-induced mechanical hyperalgesia requires PKA and PKCε activity. (A) Dosage curve for protein kinase A (PKA) and protein kinase Cε (PKCε) inhibitors. Mice received intraplantar injection with 25 μl of different doses of PKA inhibitor (PKAI, H89) before 50% CFA injection or PKCε inhibitor (PKCεI, TAT-PKCεV_1-2_) or control peptide (ctrl50) at 4 h after CFA injection. The PWT on ipsilateral side was measured at 90 min after the second injection. Data are mean±SEM of total tested mice (n = 6–12 per group). ##p<0.01, ###p<0.001 compared to PKAI/CFA with CFA only group and ***p<0.001 compared to PKCεI/CFA with CFA only group by one-way ANOVA with a post-hoc Bonferroni test. (B, C) Mice were injected with PKAI (50μM) or PKCεI (50μM) before (0 h) or after (3, 4, 5 h or 1 or 16 days) CFA injection. The PWT on the ipsilateral side (B) or contralateral side (C) was measured at 90 min after the second injection. Data are mean±SEM of total tested mice (n = 6 per group). ###***p<0.001 compared to CFA-injected groups by two-way ANOVA with a post-hoc Bonferroni test. (D) Mice were injected with PKAI (50μM) or PKCεI (50μM) before (0 h) or after (3, 4, 5 h or 1 or 10 days) carrageenan injection. The PWT on the ipsilateral side was measured at 90 min after the second injection. Data are mean±SEM of total tested mice (n = 6 per group). ###***p<0.001 compared to carrageenan-injected groups by two-way ANOVA with a post-hoc Bonferroni test.

To examine the PKA and PKCε requirement, mice were injected with the inhibitor of PKA (H89, PKAI) at 90 min after CFA injection or the inhibitor of PKCε (PKCI) or control peptides (ctrl50) at 4 h after CFA injection and underwent behavior tests at 90 min after the second injection. Both 20 and 50 μM effectively ameliorated CFA-induced hyperalgesia (Fig 2A). PKCI control peptides (50 μM) did not affect the hyperalgesia (Fig 2A). The doses of 50 μM for both inhibitors were used in the following experiments. Mice were injected with PKA or PKCε inhibitor at different times before (0 h) or after (3, 4, 5 h or 1 or 16 days) CFA injection and underwent behavioral tests at 90 min after the second injection (inhibitor injection). Injection of PKA inhibitor (PKAI) before CFA injection reduced the response to mechanical stimuli bilaterally (1.43±0.10 and 3.67±0.67 g PWT on ipsilateral and contralateral paws, respectively, as compared with the CFA control (0.6±0 and 2.0±0 g). Ameliorated hyperalgesia was observed at 3 and 4 h but not 5 h or 1 or 16 days after CFA injection (Fig 2B and 2C). PKA activity may be required early during CFA-induced hyperalgesia (<5 h). Injection of PKCε inhibitor (PKCεI) did not inhibit mechanical hyperalgesia in the first 3 h but rather at 4 h after CFA injection and lasted for 16 days (Fig 2B and 2C). PKCε activity may be involved later in CFA-induced hyperalgesia (>3 h). Similar results were found with carrageenan-induced hyperalgesia. PKA activity lasted for only 3 h and PKCε activity began at 4 h and remained up to 10 days (Fig 2D).

**Fig 3:**
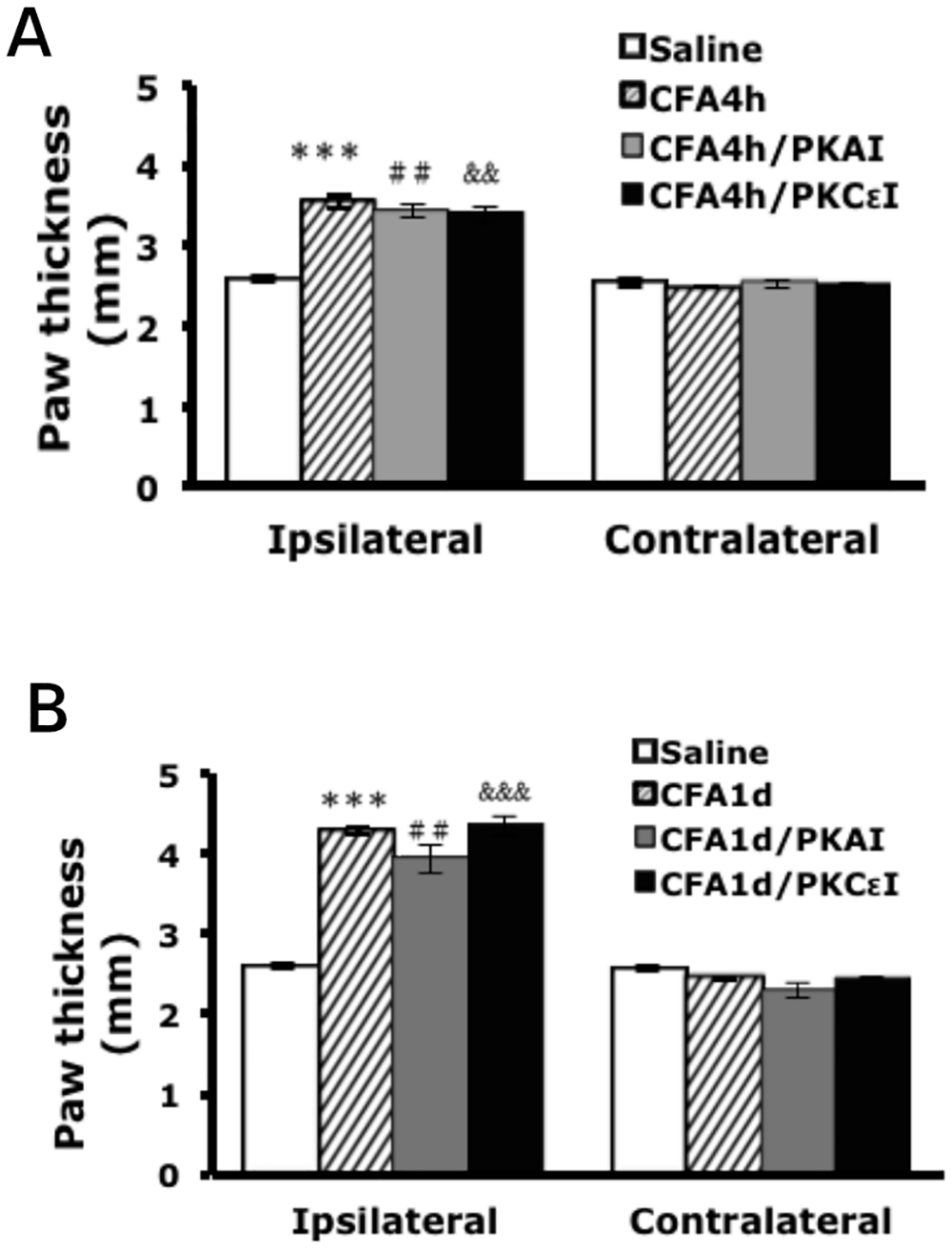
Thickness of mouse paw remains unchanged after blocking PKA or PKCε. Mice received intraplantar injection with 50μM of PKAI (H89) or PKCεI (TAT-PKCεV_1-2_) at 4 h or 1 day after 50% CFA injection. At 90 min after inhibitor injection, mice were killed and the thickness of injected (ipsilateral) and uninjected (contralateral) paws was measured. Data are presented as meanSEM of total tested mice (n = 3). Comparisons between CFA-injected (*), CFA/PKAI (#), or CFA/PKCεI (&) and saline-injected animals were analysed by one-way ANOVA with a post-hoc Bonferroni test. ##&&p<0.01, ***&&&p<0.001.

CFA injection induced unilateral peripheral edema at 4 h after injection (3.55±0.08 mm for the ipsilateral paw, 2.49±0.01 mm for the contralateral paw vs 2.58±0.01 and 2.54±0.02 mm, respectively, for the saline control) (Fig 3A). The edema peaked at 24 h after injection (4.28±0.05 mm) (Fig 3B), then gradually decreased but remained for at least 3 weeks (data not shown). Injection of PKAI or PKCεI did not reduce edema induced by CFA injection (Fig 3A and 3B), so PKA or PKCε activity may not be required for development of edema.

#### Prolonged hyperalgesia requires a switch of G_s_ and G_i_ protein dependency

**Fig 4:**
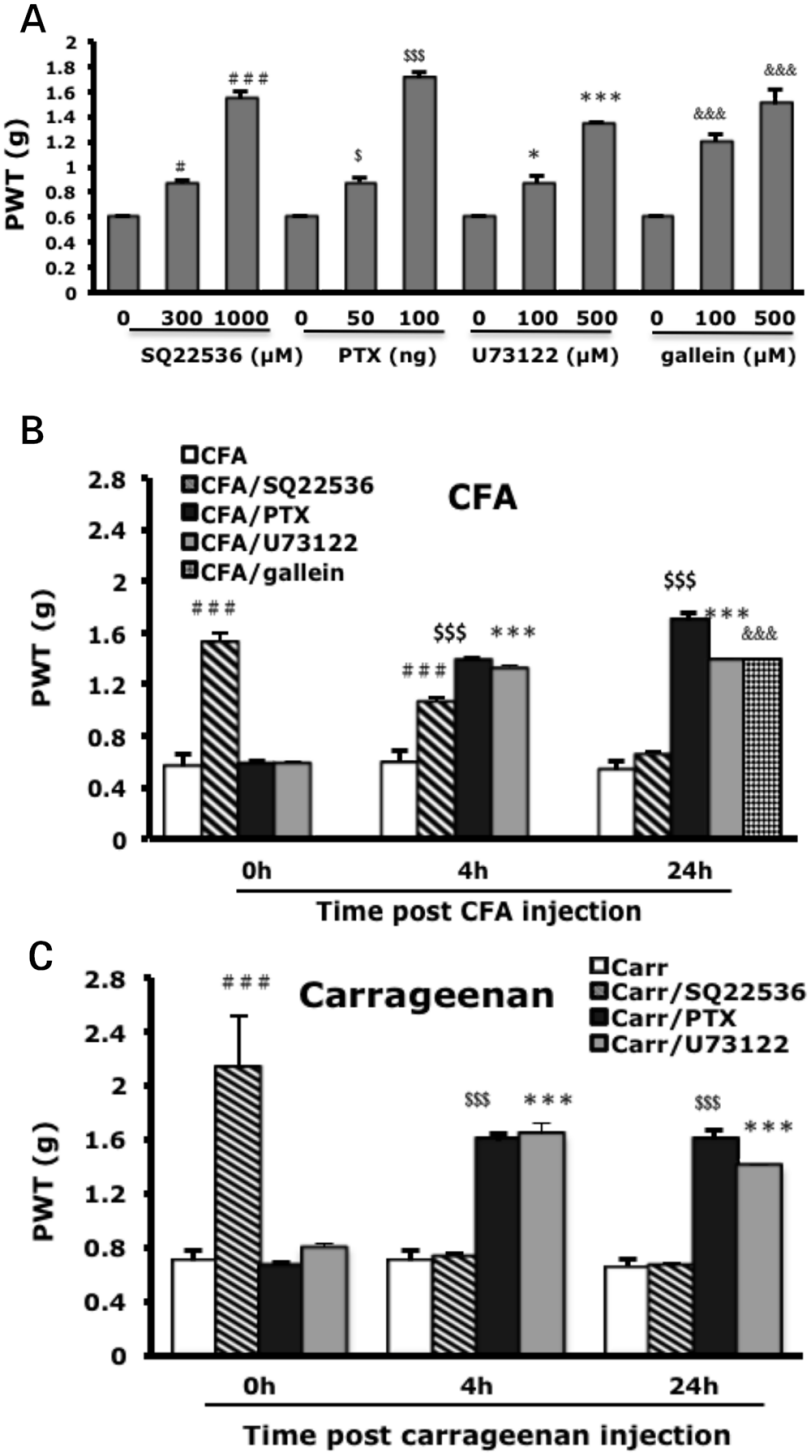
Blocking of G-protein signalling reduces CFA and carrageenan-induced mechanical hyperalgesia. (A) Mice were injected with 25 μl of AC inhibitor (SQ22536) before CFA injection or with G_i_ inhibitor (pertussis toxin [PTX]), PLC**β** inhibitor (U73122), or G_βγ_ inhibitor (gallein) 1 day after CFA injection. Data are mean±SEM of total tested mice (n = 6–12 per group). #$*p<0.05, &&P<0.01, $$$###&&&***p<0.001 compared to no inhibitor-injected groups by one-way ANOVA with a post-hoc Bonferroni test. (B-C) Mice were injected with AC inhibitor (SQ22536, 1 mM), G_i_ inhibitor (PTX, 100 ng) or PLC**β** inhibitor (U73122, 500 μM), before (0 h) or 4 h or 1 day after CFA (B) or carrageenan (C) injection. The threshold of paw withdrawal was measured at 90 min after the second injection. Data are mean±SEM of total tested mice (n = 6–8 per group). $$$###&&&***p<0.001 compared to CFA-injected or carrageenan-injected groups by two-way ANOVA with a post-hoc Bonferroni test.

To understand whether prolonged hyperalgesia requires G-proteins, we tested several inhibitors: SQ22536 inhibits the activity of adenylyl cyclase (AC), which is activated by G_s_ protein; pertussis toxin (PTX) blocks G_i_ protein-mediated signalling; U73122 inhibits phospholipase Cβ (PLCβ), which is activated by G_q_ or G_i_ protein; and gallein blocks G_βγ_ function. SQ22536 was injected before CFA injection and PTX, U73122, and gallein at 24 h after CFA injection. Behavioral tests were performed at 90 min after the second injection. All inhibitors reduced CFA-induced mechanical hyperalgesia in a dose-dependent manner. The doses of 1 mM SQ22536, 100 ng PTX, 500 μM U73122, and 500 μM gallein were used for following experiments (Fig 4A). SQ22536 inhibited CFA-induced mechanical hyperalgesia early after injection (0–4 h), which corresponds to the effective time of PKAI (Figs 4B and 2B). U73122, PTX, and gallein had inhibitory effects after 4 h (Figs 4B and 2B), which agreed with the PKCεI effective time. Similar results were found in the carrageenan model. The effective time for SQ22536 was 0–3 h and >3 h for U73122 and PTX (Figs 4C and 2D). The G_s_-PKA pathway may act on the early phase of prolonged hyperalgesia and the G_iβγ_-PKCε pathway the late phase.

#### Acute hyperalgesia induced by PGE_2_ or 5-HTTHTHThHcc requires PKA or PKCε activity

**Fig 5:**
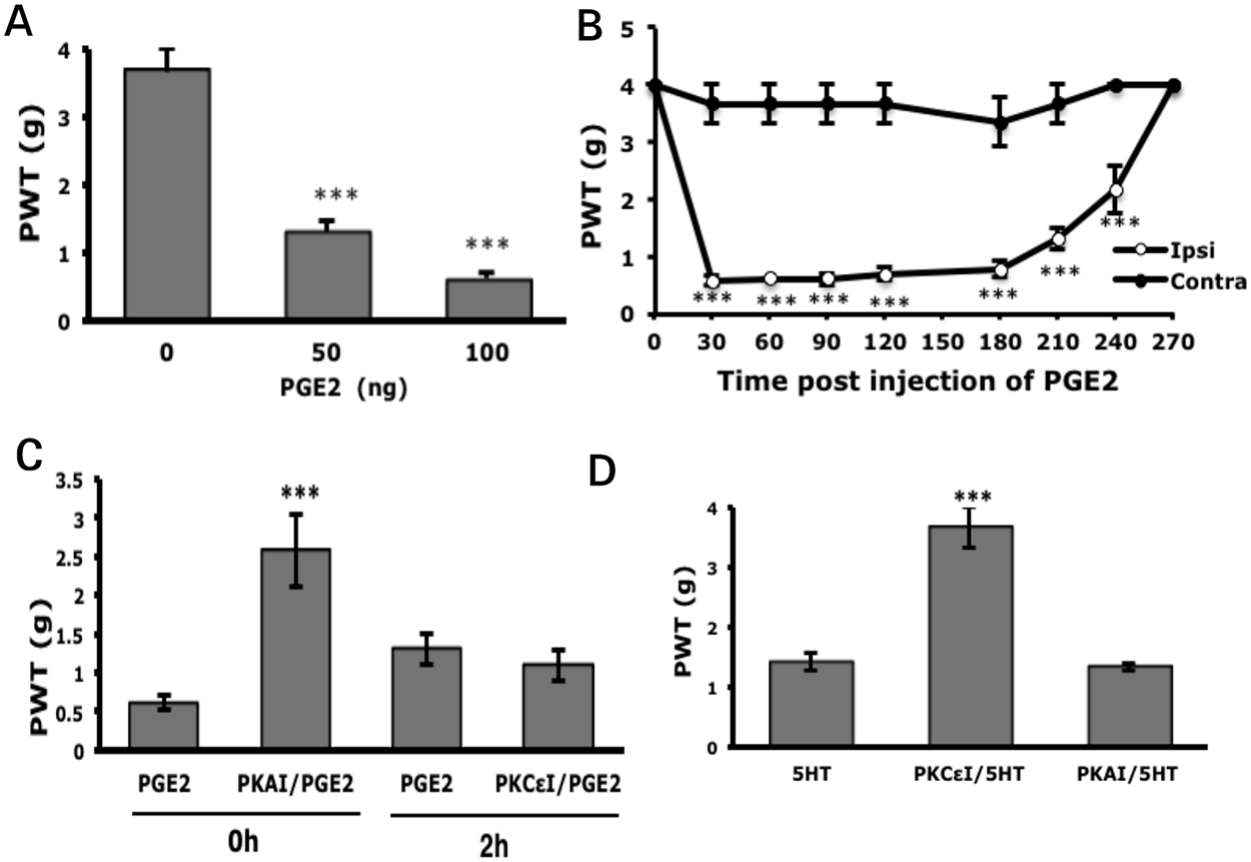
Blocking PKA or PKCε reduces PGE_2_ or 5-HT-induced mechanical hyperalgesia, respectively. (A) Mice were intraplantarly injected with 25 μl PGE_2_, followed by mechanical test at 90 min after injection. ***p<0.001 compared with saline-injected by one-way ANOVA with a post-hoc Bonferroni test. (B) Mice were injected with PGE_2_ (100 ng). The threshold of paw withdrawal was measured before (t = 0) and after injection. ***p<0.001 compared to contralateral side by two-way ANOVA with a post-hoc Bonferroni test. (C) Mice were injected with PKAI (50μM, H89) before PGE_2_ (100 ng) injection or with PKCεI (50μM, TAT-PKCεV1-2) 2 h after PGE_2_ injection. The threshold of paw withdrawal was measured at 90 min after the PGE_2_ injection. ***p<0.01, compared to PGE_2_-injected group by one-way ANOVA with a post-hoc Bonferroni test. (D) Mice were pre-injected with PKAI (50μM) or PKCεI (50μM) before 5-HT (10μM) injection, followed by measurement of the threshold of paw withdrawal at 30 min after 5-HT injection. ***p<0.001 compared with 5-HT-injected by one-way ANOVA with a post-hoc Bonferroni test. Data are mean±SEM of total tested mice (n = 6–10 per group).

We further examined whether acute hyperalgesia induced by injection of a single inflammatory mediator (PGE_2_, 5-HT, or proton) depends on PKA and PKCε. PGE_2_ (50 ng) conferred 1.3±0.16 g PWT on ipsilateral paws, and 100 ng conferred lower PWT (0.57±0.10 g) (Fig 5A). Therefore, 100 ng PGE_2_ was used for the following experiments. Injection of PGE_2_ (100 ng) caused short-term, unilateral hyperalgesia immediately at 30 min (0.57±0.10 and 3.67±0.33 g PWT on ipsilateral and contralateral paws, respectively) and hyperalgesia lasted for 4.5 h (Fig 5B). Pre-injection of PKAI before PGE_2_ injection reduced PGE_2_-induced hyperalgesia (0.57±0.10 vs 2.57±0.46 g PWT, Fig 5C). Pre-injection of PKCεI did not prevent PGE_2_-induced hyperalgesia (data not shown). Given that the inhibitory effect of PKCεI occurred after CFA injection, we, thus, injected PKCεI at 2 h post PGE_2_ injection and found no reduction in PGE_2_-induced hyperalgesia (1.10±0.19 vs 1.30±0.19 g PWT, Fig 5C). Thus, only PKA activity may be involved in PGE_2_-induced acute hyperalgesia, which agrees with a previous study [16]. Injection of 5-HT induced acute hyperalgesia in our previous study [25]. Hyperalgesia induced by 5-HT depended on only PKCε because PKCεI but not PKAI specifically inhibited hyperalgesia (Fig 5D).

#### Acute hyperalgesia induced by acidic solutionTHTHThHcc requires both PKA and PKCε activity

**Fig 6:**
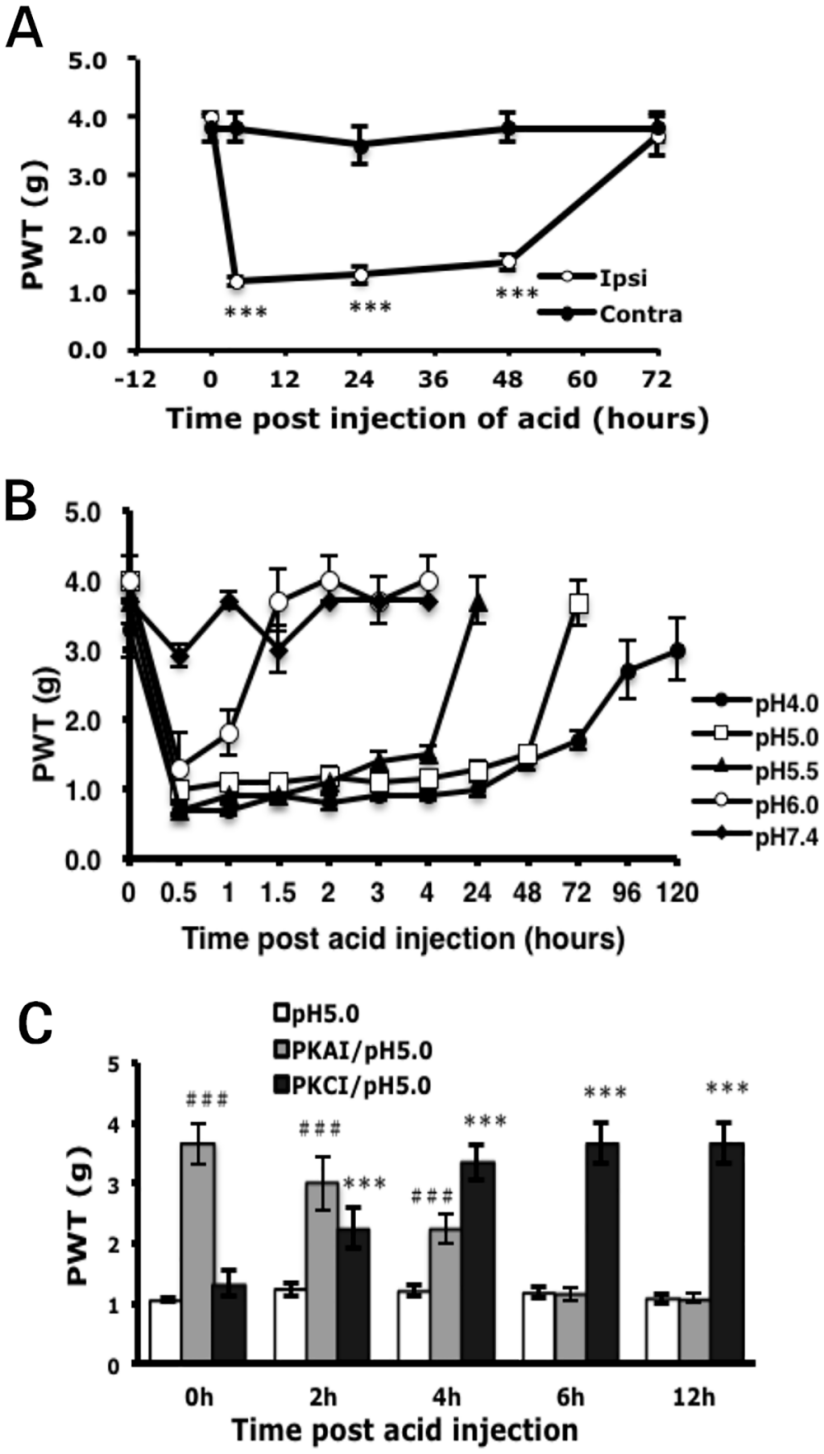
Acid-induced mechanical hyperalgesia requires PKA and PKCε activity. (A) Mice received intraplantar injection with 25 μl of acid solution (pH 5.0). The threshold of paw withdrawal was measured before (t = 0) and after injection. ***p<0.001 compared to contralateral side by two-way ANOVA with a post-hoc Bonferroni test. (B) Mice were injected with 25 μl acid solutions (pH 7.4, 6.0, 5.5, 5.0, 4.0). The threshold of paw withdrawal was measured before (t = 0) and after injection. (C) Mice were injected with PKAI (50μM) or PKCεI (50μM) before (0 h) or at 2, 4, 6, or 12 h after acid (pH 5.0) injection. The threshold of paw withdrawal was measured at 60 min after the second injection. Data are mean±SEM of total tested mice (n≥6 per group). ***###p<0.001 compared with acid-injected by two-way ANOVA with a post-hoc Bonferroni test.

Administration of acidic solution (pH 5.0) induced unilateral hyperalgesia at 30 min, with 1±0.08 g PWT on ipsilateral paws (Fig 6A). Although the PWT was not as low as with PGE_2_ injection (Fig 5B), acidic pH-induced hyperalgesia lasted longer, for 2 days. The duration of hyperalgesia depended on the pH. With pH 6.0 solution injection, hyperalgesia lasted for only 1 h. Hyperalgesia induced by pH 5.5 solution was maintained for 4 hours, and 3 days’ longer hyperalgesia was found with strong acidic solution (pH 4.0) (Fig 6B). We then tested whether PKA or PKCε is involved in acid-induced hyperalgesia. Administration of PKAI before injection of acidic solution (pH 5.0, 0 h) or 2 or 4 h after injection reduced acid-induced hyperalgesia (3.67±0.33 vs 1.06±0.05 g PWT at 0 h; 3.00±0.44 vs 1.25±0.13 g at 2 h; 2.25±0.25 vs 1.25±0.13 g at 4 h, Fig 6C) but not at 6 or 12 h. Administration of PKCεI at 2, 4, 6, or 12 h after injection of acidic solution, but not pre-injection of PKCεI, reduced acid-induced hyperalgesia (Fig 6C). Although both PKA and PKCε are involved in acid-induced acute hyperalgesia, PKA regulated hyperalgesia at 0 to 4 h and PKCε was responsible for the regulation after 2 h. Similar results were observed with injection of pH 5.5 solution: the time for the switch of PKA and PKCε dependence was about 2 to 4 h after injection (data not shown).

#### Acid signals induce PKA and PKCε translocation

**Fig 7:**
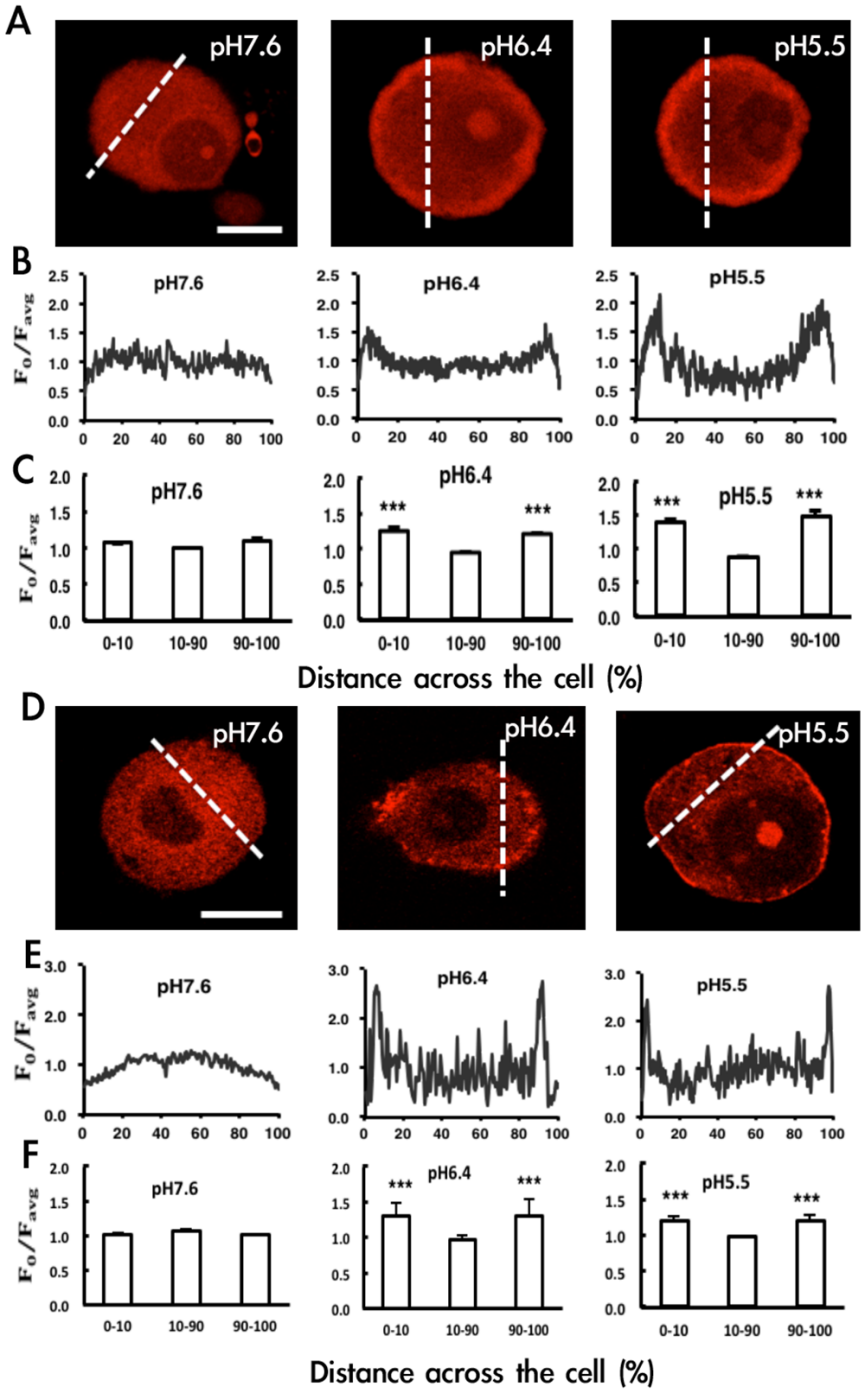
Acid induces PKA and PKCε translocation. Primary dorsal root ganglia (DRG) from CD1/ICR mice were cultured for 12~16 h, then stimulated with acid solution, pH 7.6, 6.4 or 5.5 for 5 or 30 min or 15 s, respectively. After immunostaining with anti-PKA or anti-PKCε antibodies, cell images with red fluorescence for PKA (A) or PKCε (D) were observed by confocal microscopy. Bar = 10μm. (B, C, E, F) Fluoresence intensity (F_0_) in a line bisecting the neuronal soma (see dashed line in A, D) was measured and the profile of intensity (F_0_/F_avg_) is shown in B, E. F_avg_ = the mean intensity of the dashed line. Lines were positioned to avoid nuclei. Fluorescence intensity within the peripheral (0–10% and 90–100% of cell distance) region was the membrane fraction and within the central region (10–90% of cell distance) the cytosolic fraction. Shows the mean intensity of membrane or cytosolic fractions (n = 3) (C, F). ***P<0.001 compared to cytosolic fraction (10–90%) by one-way ANOVA with a post-hoc Bonferroni test.

To understand whether acid signals can activate PKA or PKCε, we examined PKA and PKCε translocation in cultured DRG neurons after stimulation with acidic solution (pH 7.6, 6.4, and 5.5). At pH 7.6, immunoreactive PKA was predominantly located in the cytosol but was translocated to the plasma membrane at pH 6.4 and 5.5 (Fig 7A). The intensity of fluorescent signals in the central region (cytosol, 10–90% distance) and peripheral region (membrane, 0–10% and 90–100% distance) of soma was quantified (Fig 7B). The mean fluorescence intensity in the membrane fractions was equal to that in the cytosolic fraction at pH 7.6, with increased intensity in the membrane fraction found at pH 6.4 and 5.5 (Fig 7C), so PKA may have translocated from the cytosol to the membrane. Similar results were found for PKCε (Fig 7D, 7E and 7F). The PKCε signal was most intense in the cytosol at pH 7.6 and was increased in the membrane fraction at pH 6.4 and 5.5.

#### Proton-sensing GPCR genes are involved in prolonged hyperalgesia

**Fig 8:**
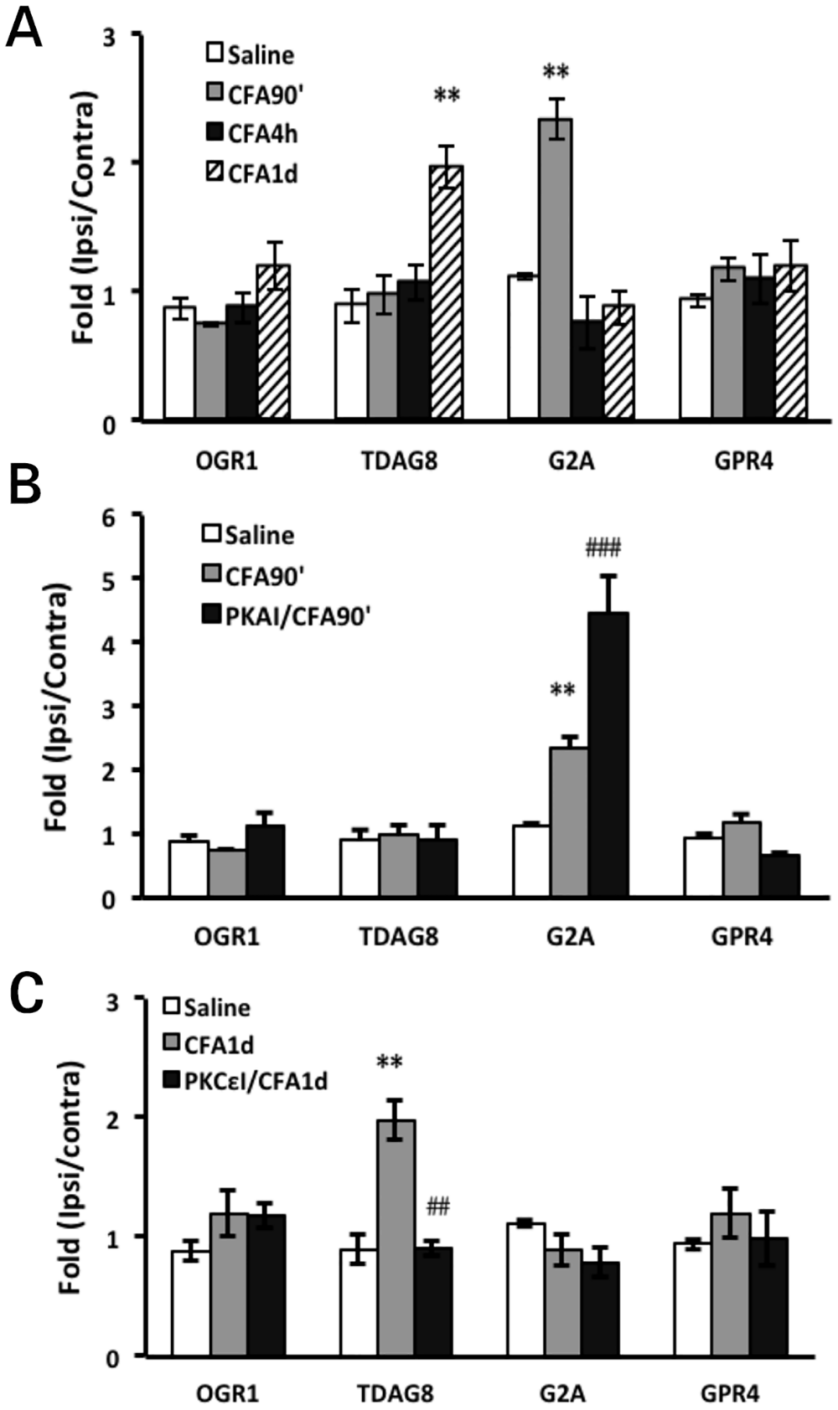
Gene expression patterns of OGR1 family after inhibition of PKA or PKCε activity. Mice received intraplantar injection with 25 μl CFA (A), with pre-injection with PKAI (B) or post-injection with PKCεI 1 day after CFA injection (C). At 90 min and 4 and 24 h after CFA injection or at 90 min after the second injection, mice were killed. RNA was obtained from lumbar 4–6 DRG ipsilateral and contralateral to injected paws for RT-PCR. The expression of each gene on the ipsilateral DRG was normalized to mGAPDH expression, then represented as relative expression to contralateral controls (fold change in expression). Data are mean±SEM of triplicate measurements (n = 3 mice). **p<0.01 compared to saline-injected group by one-way ANOVA with a post-hoc Bonferroni test. ##p<0.01, ###p<0.001 compared to CFA-injected group by one-way ANOVA with a post-hoc Bonferroni test.

Hyperalgesia induced by acidic solution (proton) but not PGE_2_ or 5-HT requires PKA and PKCε activity, and the switch time of kinase dependency is about 2 to 4 h (Figs 5C, 5D and 6C), which is similar to CFA- or carrageenan-induced prolonged hyperalgesia (Figs 2B and 2D). Acidosis result from CFA or carrageenan injection may mediate PKA and PKCε dependency. Given that G-protein signalling is also required for the development of hyperalgesia (Figs 4B and 4C), proton-sensing GPCRs may participate in the switch of PKA and PKCε dependency. Thus, we examined the expression of proton-sensing GPCRs in the CFA model. DRG were analyzed for gene expression at 90 min and 4 and 24 h after CFA injection. G2A expression was increased (2.26±0.37-fold) at 90 min after CFA injection and TDAG8 expression (1.95±0.31-fold) at 1 day after injection (Fig 8A), which is similar to previous findings [27]. OGR1 and GPR4 showed no alterations in expression. After injection of PKAI, G2A expression was further increased (4.38±0.69-fold, 50% increase, Fig 8B) at 90 min, which suggests that PKA inhibited the upregulation of G2A expression. In contrast, inhibition of PKCε activity reduced TDAG8 expression to basal levels at 1 day (Fig 8C), which suggests that PKCε activity increased TDAG8 expression. G2A may have anti-nociceptive function to prevent development of hyperalgesia, whereas TDAG8 could have a pro-nociceptive role in the development of hyperalgesia.

## Discussion

In this study, we have demonstrated that prolonged hyperalgesia in mice induced by CFA and carrageenan was regulated by PKA and PKCε. The switch time for PKA and PKCε dependency was about 3 to 4 h and the longer time for PKA dependency seemed to be associated with longer hyperalgesia. Acute hyperalgesia induced by PGE_2_ or 5-HT was regulated by PKA or PKCε, respectively. However, acute hyperalgesia induced by acidic solution (pH 5.5 or 5.0) depended on both PKA and PKCε, as for prolonged hyperalgesia induced by CFA or carrageenan. The switch time for PKA and PKCε dependency was about 2 to 4 h. Therefore, the switch of PKA and PKCε dependency in prolonged hyperalgesia induced by CFA or carrageenan could be due to acidosis signals. CFA increased G2A transcript levels at 90 min and TDAG8 transcript levels at 1 day. PKA inhibition further enhanced G2A expression, but blocking PKCε reduced TDAG8 expression. G2A may have an anti-nociceptive role and TDAG8 a pro-nociceptive role in the development of hyperalgesia.

Both carrageenan and CFA caused prolonged hyperalgesia, but hyperalgesia induced by carrageenan was unilateral and shorter (reduced to baseline levels at 16 days in the carrageenan model and 28 days in the CFA model) (Fig 1A and 1B). Unilateral CFA injection (12.5 μg) induced bilateral mechanical hyperalgesia, which agrees with previous results [28,29]. Decaris et al. [30] found that a low dose of CFA injection (1 μg) led to local inflammation (characterized by edema) without systemic effects (the absence of febrile response and IL-6 production), but a high dose (1 mg) induced systemic effects. Thus, the bilateral mechanical hyperalgesia seen in our studies could be due to systemic effects induced by the dose of 12.5 μg CFA used. In contrast, unilateral injection of carrageenan (even at 500 μg) induced only unilateral hyperalgesia. This difference could be due to the doses used or the agent itself (induce different mechanisms).

Intraplantar injection of PKA or PKCε inhibitor reduced CFA-induced hyperalgesia bilaterally but had no effect on edema (Figs 2B, 3A and 3B). Thus, PKA and PKCε could act directly on the peripheral nociceptive signals resulting in mechanical hyperalgesia and spread of pain (contralateral pain) but not inflammation. PKA and PKCε more likely alter pain thresholds to mechanical stimuli. PKC activation may increase mechanically activated currents by inducing insertion of mechanosensitive channels [31]. PKA activation has no effect on mechanical activated currents, but when applied with the PKC activator, had a synergistic effect in increasing action potential firing rates [31], which could be due to modulation of the tetrodotoxin—resistant voltage-gated sodium channels by PKA [32]. Accordingly, PKA-mediated events may act on neuronal excitability, whereas PKCε-mediated events could increase levels of mechanosensitive channels. This observation could explain in part why PKA is essential in the early phase and PKCε in the later phase of prolonged hyperalgesia.

PKA activity was required from the beginning to 3 or 4 h in the carrageenan or CFA model, respectively (Fig 2B and 2D). PKCε activity appeared from 4 h to the end of hyperalgesia (Fig 2B and 2D), which is consistent with the results of priming studies [17,18]. Parada et al. [19] previously suggested that cAMP could stimulate some mechanisms required for ongoing PKCε activity. In contrast, several lines of evidence demonstrated that PLCβ3 attenuates carrageenan-induced hyperalgesia and prevents carrageenan-induced priming [33]. G_i_ could be involved in stress or inflammation-induced priming [21], and opioid-induced hyperalgesic priming is mediated by G_i_ and PKCε [23, 34]. CCL2 increased Na_v_1.8 channel activity via a G_βγ_-dependent pathway [35]. We found that the AC inhibitor (SQ22536) decreased hyperalgesia in the beginning (<4 h), and the G_i_ protein inhibitor (PTX), PLCβ inhibitor (U73122), and G_βγ_ inhibitor (gallein) reduced hyperalgesia from 4 h after CFA or carrageenan injection (Fig 4B and 4C). Thus, the G_s_-AC-PKA pathway may be responsible for the early phase of hyperalgesia and G_iβγ_-PLCβ-PKCε pathway for the late phase.

Although PKA activity was required in the first 3 or 4 h, it may affect the duration of hyperalgesia. CFA-induced hyperalgesia was longer than carrageenan-induced hyperalgesia. PKCε activity appeared from 4 h in both models, but PKA activity was maintained for 4 h in CFA model, and 3 h in the carrageenan model. The longer the PKA activity is maintained, the longer the hyperalgesia seems to last. The G_s_-AC-PKA pathway may trigger some mechanisms to activate the G_iβγ_-PLCβ-PKCε pathway.

PGE_2_ and 5-HT induced transient hyperalgesia, which depended on PKA and PKCε, respectively (Fig 5C and 5D). Protein kinase requirements for acute hyperalgesia may depend on the inflammatory mediator used for induction. 5-HT-induced mechanical hyperalgesia is mediated by the 5-HT_2B_ receptor and 5-HT_2B_ activates the G_q_-PLCβ-PKC pathway [25], which may explain why 5-HT-induced mechanical hyperalgesia depends on PKCε activity (Fig 5D). EP4, coupled to the G_s_-PKA pathway, may be involved in CFA-induced inflammatory pain [36]. EP4 may also mediate PGE_2_-induced mechanical hyperalgesia that requires PKA activity.

Hyperalgesia induced by acidic solution such as CFA and carrageenan required PKA and PKCε for the initiation and maintenance of hyperalgesia, and the switch time for PKA/PKCε dependency was about 2 to 4 h. Steen et al. [11] proposed that acidosis in inflamed tissues is the decisive factor inducing pain and hyperalgesia, whereas a combination of inflammatory mediators plays a role in sensitizing the low pH effect. Therefore, the switch of PKA and PKCε dependency in our CFA and carrageenan models could be due to the acidosis. Indeed, we found that acid signals induced PKA and PKCε translocation in cultured neurons, which suggests that acid signals can activate PKA and PKCε. Both mild acid (pH 6.4) and strong acid (pH 5.5) resulted in significant translocation of PKA and PKCε.

Acid signals induce the translocation of PKA and PKCε, possibly through proton-sensing GPCRs. GPR4 and TDAG8, which are coupled with the cAMP-PKA pathway through G_s_ protein [27,37,38], are potential candidates for PKA activation. After CFA injection, GPR4 gene expression did not change, but TDAG8 expression increased two-fold at day 1. Inhibition of PKCε but not PKA reduced TDAG8 expression to basal levels. Spinal TDAG8 and downstream PKA signalling contribute to bone cancer pain [39]. Peripheral TDAG8 likely mediates downstream PKA signalling to promote the development of hyperalgesia, and increased TDAG8 expression is required for prolonged hyperalgesia. PKCε activity enhanced TDAG8 expression to maintain prolonged hyperalgesia. Given that TDAG8 can sensitize TRPV1 function [27] and TRPV1 is involved in mechanical hyperalgesia [40–44], TDAG8 may regulate TRPV1 function to facilitate development and maintenance of hyperalgesia. Several studies suggested that TDAG8 in macrophages or microglia regulates cytokine production to attenuate inflammation [45–47], which may explain the anti-inflammatory role of TDAG8. Whether TDAG8 has positive or negative effects on inflammation-induced hyperalgesia remains for further exploration.

At 90 min after CFA injection, G2A expression was increased two-fold. Inhibition of PKA, but not PKCε further enhanced G2A expression. In endothelial cells, G2A expression inhibits macrophage accumulation by blocking NF-kB activation and chemokine expression [48]. G2A signalling in macrophages or phagocytes may facilitate efferocytosis of dying cells, thus preventing ongoing inflammation [49,50]. G2A expression likely increases in the beginning of inflammation to inhibit macrophage accumulation, and PKA signalling inhibits G2A expression to promote the development of inflammation and hyperalgesia. Therefore, G2A may play an anti-nociceptive role.

## Conclusions

This study demonstrated that the switching of PKA and PKCε dependency in hyperalgesia induced by inflammation and acidosis. G_s_-AC-PKA signalling contributed to the early phase of hyerpalgesia while G_iβγ_-PLCβ-PKCε to the later phase. Acidosis signal could be one of the decisive factors for the switching of PKA and PKCε dependency, possibly through the proton-sensing GPCRs, TDAG8 and G2A.
